# Safety and efficacy of the immunosuppressive agent 6-tioguanine in murine model of acute and chronic colitis

**DOI:** 10.1186/1471-230X-11-47

**Published:** 2011-05-05

**Authors:** Miloslav Kverka, Pavel Rossmann, Helena Tlaskalova-Hogenova, Klara Klimesova, Bindia Jharap, Nanne K de Boer, Rene M Vos, Adriaan A van Bodegraven, Milan Lukas, Chris J Mulder

**Affiliations:** 1 Department of Immunology and Gnotobiology, Institute of Microbiology, Academy of Sciences of the Czech Republic, Videnska 1083, 14220 Prague 4, Czech Republic; 2 Department of Gastroenterology and Hepatology, VU University Medical Center, P.O. Box 7057, 1007 MB Amsterdam, The Netherlands; 3 Department of Clinical Pharmacology and Pharmacy, VU University Medical Center, P.O. Box 7057, 1007 MB Amsterdam, The Netherlands; 4 Clinical and Research Center for Inflammatory Bowel Disease ISCARE-Lighthouse, Jankovcova 1569/2c, 170 04 Prague 7, Czech Republic

## Abstract

**Background:**

Oral thiopurines are effective and widely used in treatment of inflammatory bowel disease (IBD) in humans, although their use is limited due the development of adverse events. Here, we examine the efficacy and toxicity of oral treatment with 6-tioguanine (6-TG) and azathioprine (AZA) in a murine model of IBD.

**Methods:**

We induced acute or chronic colitis in BALB/c mice by one or four cycles of 3% dextran sulphate sodium (DSS), respectively. Mice were treated by daily gavages of various dosages of 6-tioguanine, azathioprine, or by phosphate buffered saline (PBS) starting the first day of DSS or after two cycles of DSS, respectively. We monitored the efficacy and toxicity by measuring the weight change and serum alanine aminotransferase (ALT) activity and by disease severity and histology, at the end of the experiment. Moreover, we measured cytokine production after colon fragment cultivation by enzyme-linked immunoabsorbent assay and numbers of apoptotic cells in the spleen by flow cytometry.

**Results:**

6-TG is effective in the treatment of acute DSS-induced colitis in a dose-dependent manner and 40 μg of 6-TG is significantly more effective in the treatment of acute colitis than both AZA and PBS. This effect is accompanied by decrease of IL-6 and IFN-γ production in colon. We did not observe histological abnormalities in liver samples from control (PBS) or 6-TG treated mice. However, liver samples from most mice treated with AZA showed mild, yet distinct signs of hepatotoxicity. In chronic colitis, all thiopurine derivatives improved colitis, 20 μg of 6-TG per dose was superior. High doses of 6-TG led to significant weight loss at the end of the therapy, but none of the thiopurine derivatives increased levels of serum ALT. Both thiopurine derivatives reduced the proportion of apoptotic T helper cells, but a high production of both IL-6 and TGF-β was observed only in colon of AZA-treated mice.

**Conclusions:**

Use of 6-TG in the treatment of experimental colitis in mice appears superior to AZA administration and placebo. In contrast to 6-TG, the use of AZA resulted in histological liver abnormalities.

## Background

The immune-modulating thiopurines, 6-mercaptopurine (6-MP), and its pro-drug azathioprine (AZA), are widely used in inflammatory bowel diseases (IBD) treatment [1-3]. Both AZA and 6-MP require extensive metabolisation before the pharmacologicaly active metabolites, 6-thioguaninenucleotides (6-TGN), are generated.

Their mechanism of action is ascribed to both cytotoxic and apoptototic pathways. Owing to their structural similarity to endogenous purine bases, 6-TGN are incorporated into DNA or RNA as fraudulent bases, ultimately leading to cytotoxicity. Activity of the specific metabolite, 6-thioguanine-triphosphate was recently found to contribute to the overall molecular immunosuppressive effect. This end-metabolite induces apoptosis and decreases the expression of proinflammatory molecules in activated T cells [4,5].

Although thiopurine derivatives are considered to be a relatively safe maintenance therapy, several studies report discontinuation of thiopurine derivatives in up to 50% of patients during long-term therapy, mainly due to the development of adverse events [6-8].

Another thiopurine, 6-tioguanine (6-TG), has been proposed as a rescue drug for IBD patients failing to tolerate or respond to AZA and 6-MP [9,10]. However, this suggestion has been discouraged, when histological liver abnormalities, in particular nodular regenerative hyperplasia (NRH), were found in 6-TG treated IBD patients [11,12]. These findings were verified by German and Austrian studies [13], but not by Irish and Dutch studies with a follow-up of 3-5 years [14-18]. Debate is ongoing whether 6-TG is potentially more hepatotoxic than other thiopurine derivatives such as conventional AZA or 6-MP, or whether its alleged hepatotoxicity is dose-dependent [19]. We therefore designed an AZA- and placebo-controlled study using different doses of 6-TG to assess the therapeutic efficacy and (hepato) toxicity in the murine model of acute and chronic dextran sulphate sodium (DSS)-induced colitis.

## Methods

### Mice

We used conventional female, 3-month-old BALB/c mice (Institute of Physiology AS CR, Prague, Czech Republic) in this study. The experiments were approved by the Institutional animal care and use committee at the Academy of Sciences of the Czech Republic.

### Experimental design

Acute colitis was induced by 3% (weight/volume) dextran sulfate sodium (DSS) (molecular weight 36-50 kDa; MP Biomedicals, Inc.) dissolved in drinking water for 9 days. Starting from the first day of DSS administration, we administered 6-TG in a daily dosage of 10 μg, 20 μg or 40 μg (obtained from Mosadex C.V., Elsloo, The Netherlands), or AZA (Imuran^® ^Glaxo-SmithKline) in a daily dosage of 30 μg or 60 μg. Both agents were dissolved in 100 μl of sterile phosphate buffered saline (PBS) and administered by daily gavage. Control group was treated only with PBS. Taking into account the average mice weight, the dose per kilogram for 10 μg, 20 μg, 40 μg of 6-TG, 30 μg or 60 μg AZA was 0.45 mg/kg, 0.91 mg/kg, 1.82 mg/kg, 1.37 mg/kg or 2.73 mg/kg, respectively.

Chronic colitis was induced by four cycles of 3% DSS (5 days DSS, 9 days water). The treatment with daily oral dose of either PBS, 20 μg of 6-TG, 40 μg of 6-TG or 60 μg AZA started after the 2^nd ^cycle of DSS, once the chronic colitis was established. Each mouse therefore received during these 22 doses a cumulative dose of either 19.8 or 39.3 mg/kg of 6-TG, or 58.1 mg/kg of AZA, respectively.

### 6-TG metabolite monitoring

To analyse the concentration of 6-tioguanine nucleotides in red blood cells, we collected blood samples at the last day of experiment in EDTA. The samples were centrifuged to isolate erythrocytes and after washing with PBS, erythrocyte counts were done. Samples were than stored at -80°C until analysis, performed as described previously [20].

### Assessment of colitis severity

Colitis was evaluated on the last day of the experiment by using a disease activity index (DAI), colon length, and histological scoring system. The DAI scores body weight loss, stool consistency, and the presence of the blood in the stool, as previously described by Cooper et al. [21]. Occult blood in faeces was evaluated with Faecal Occult Blood Test (Okult-viditest Rapid; Vidia, Vestec, Czech Republic). No deaths occurred during the experiment.

Following sacrificing, the entire colon was removed (from caecum to anus) and placed without tension on a ruler. Colon length was measured as an indirect marker of inflammation. The descending colon was fixed in 4% formalin, and stained with haematoxylin and eosin (HE) to evaluate mucosal damage during acute colitis or with Sirius red (Sigma-Aldrich) and Haematoxylin to evaluate mucosal damage in chronic colitis model. The microscopic findings in acute colitis were assessed semi-quantitatively and weighted score developed in our laboratory for each section was obtained, ranging from 0 (no signs of colitis) to 3 (severe colitis) as described previously [22]. To describe mucosal damage in chronic colitis model we used different scoring system to describe, based on the scoring system validated by Dieleman et al. [23]. Additionally, Sirius red staining was added to score collagen deposition. Briefly, we scored the grade of five parameters (inflammation, extent, regeneration, crypt damage and collagen deposition). Each of these changes was also multiplied by the percentage quantifying the disease involvement on cross sections of colon: (1) 1-25%; (2) 26-50%; (3) 51-75%; (4) 76-100%. The final score was calculated by the sum of the scores for all five parameters.

We used mouse haptoglobin enzyme-linked immunoabsorbent assay (ELISA; Alpco diagnostic, Salem, NH, USA) to analyse the concentration of acute phase protein haptoglobin, a marker of inflammation, in serum of mice with chronic colitis.

### Histological assessment of liver toxicity

Livers were fixed in 4% formalin, embedded in paraffin and stained with haematoxylin and eosin or with Gomori's silver impregnation for reticulin (4 tissue sections per mice were evaluated). Two expert liver pathologists, unaware of the treatment of the mice, were asked to meticulously evaluate the liver samples independently with special focus on the presence of sinusoidal dilatation, veno-occlusive disease, fibrosis, cirrhosis, NRH, steatosis, necrosis and cholestasis.

### Serological assessment of liver toxicity

To monitor the hepatotoxic effects in thiopurine treated animals, we measured the alanine transaminase (ALT) enzyme activity in serum by MaxDiscovery Alanine Transaminase Enzymatic Assay Kit (Bio Scientific, Austin, TX, USA) according to manufacturer's recommendations. We measured ALT activity at sacrifice and/or at the time of the 7^th^, 14^th ^and 22^nd ^dose during chronic colitis therapy.

### Analysis of cell apoptosis by Flow cytometry

Single-cell suspensions of spleen was prepared and stained for apoptotic cells using following fluorochrome labelled anti-mouse mAbs: CD3-FITC (BD Biosciences, San Jose, CA, USA), CD28-PE, CD8-PerCP.Cy5.5 (all from eBioscience, San Diego, CA, USA), CD4-Qdot^® ^605 and annexin V-Alexa Fluor^® ^647 (both Invitrogen Corp., Carlsbad, CA, USA) according to the manufacturer's recommendation. Hoechst 33258 (Sigma-Aldrich, St. Louis, MO, USA) was added just before analysis to stain for dead cells. Flow cytometric analysis was performed on LSRII (BD Biosciences), and data were analyzed using FlowJo software (Tree Star Inc., Ashland, OR, USA).

### Cytokine production

At the end of the experiment, sections of mouse colon were obtained, cut open longitudinally, washed in PBS containing penicillin and streptomycin and weighed. These tissue fragments were then cultivated for 48 h in a humidified incubator at 37°C and 5% CO_2 _in RPMI-1640 (Sigma-Aldrich) containing 10% fetal bovine serum (Biochrom AG, Berlin, Germany) and 1% Antibiotic-Antimycotic solution (Sigma-Aldrich). The supernatants were collected and stored at -20°C until analysis for cytokine production. Levels of selected cytokines were determined using commercially available ELISA sets purchased from Invitrogen (TNF-α, TGF-β, IL-10; Invitrogen Corp.) or R&D Systems (IFN-γ, IL-6; R&D Systems Inc., Minneapolis, MN, USA). All tests were performed according to the manufacturers' recommendations.

### Statistical analysis

Values are presented as mean ± standard deviation (SD). Differences in colon length, DAI and histological score, cell populations, ALT activity and haptoglobin levels of multiple groups were compared with one-way analysis of variance (ANOVA) with Tukey's multiple comparison test. Differences in weight change during time of multiple groups were compared to the control group (PBS/DSS) by two-way repeated measure ANOVA with Bonferroni's post-hoc test. Differences were considered statistically significant at P < 0.05. GraphPad Prism statistical software (version 5.03, GraphPad Software, Inc. La Jolla, CA, USA) was used for analyses.

## Results

### 6-TG is more effective and less toxic than AZA in acute colitis model

**Figure 1:**
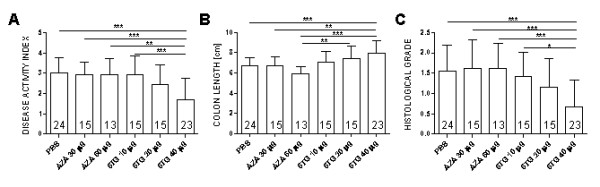
**Evaluation of treatment efficacy in acute DSS-induced colitis**. Values are expressed as mean (bar) ± standard deviation (whisker). (A) DAI; (B) Colon length (cm) and (C) Histological grade. The differences among experimental groups are analyzed with one-way analysis of variance (ANOVA) with Tukey's multiple comparison test (*P < 0.05; **P < 0.01; ***P < 0.001). Data are pool of four independent experiments; numbers in bars indicate number of animals per group.

**Figure 2:**
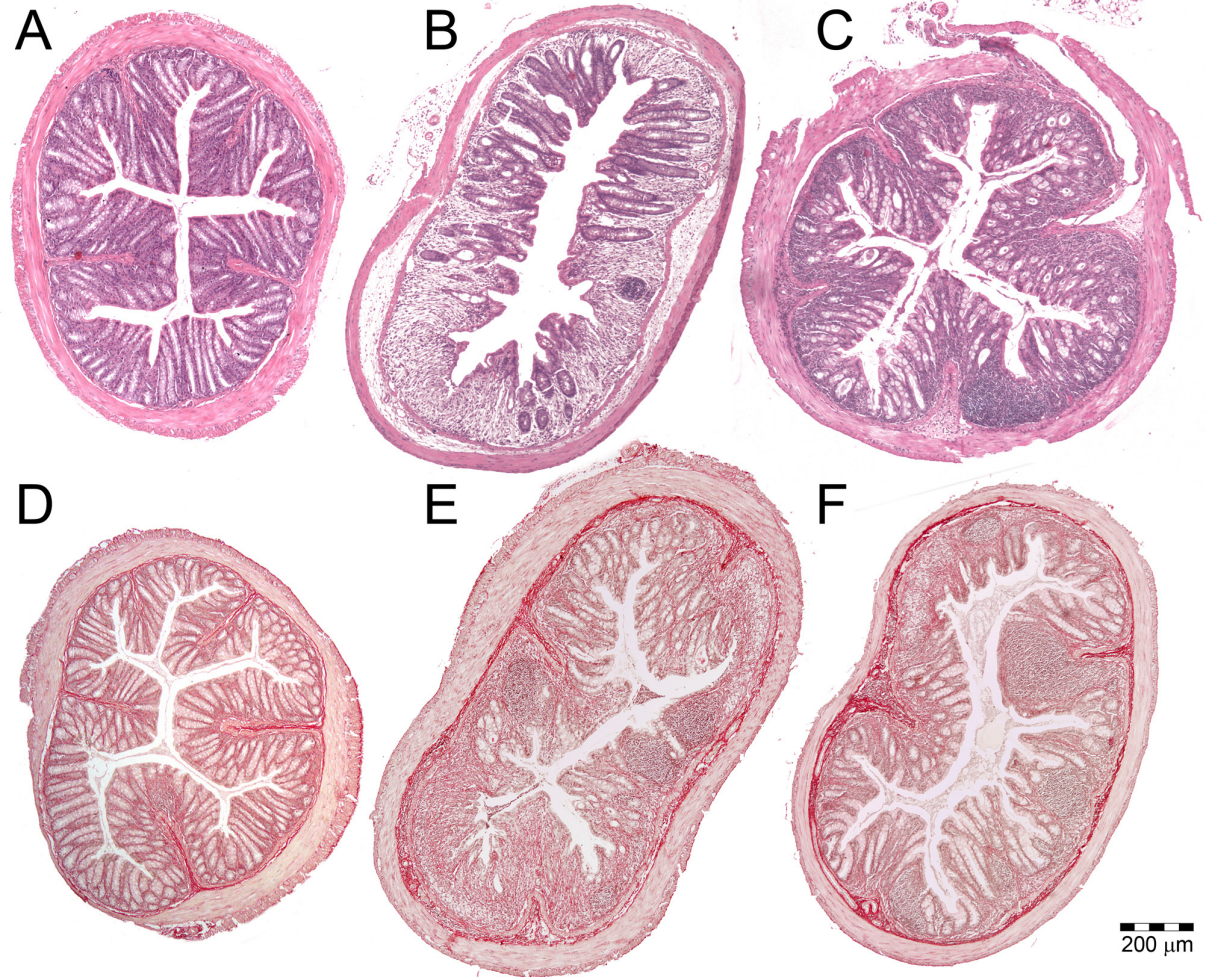
**Evaluation of treatment efficacy by colon histology**. HE stained slides showing A: Healthy colon (score 0.0); B: severely inflamed colon (score 2.0) from the control group during acute colitis; C: colon with medium-grade acute colitis (score 1.0) representing 6-TG treated mice. Sirius red and Haematoxylin stained slides showing D: Healthy colon (score 0); E: colon with severe chronic colitis (score 32) from the control group; F: colon with medium-grade chronic colitis (score 20) representing 6-TG treated mice

The dosage of 40 μg of 6-TG daily was significantly more effective in the treatment of acute DSS colitis than either AZA 30 or AZA 60 μg or PBS (Figure 1A-C and Figure 2A-C). The efficacy of 6-TG therapy correlates with 6-TG dosage, the mice treated with 40 μg of 6-TG had significantly longer colons and lower DAI than those treated with 10 μg of 6-TG per day.

**Figure 3:**
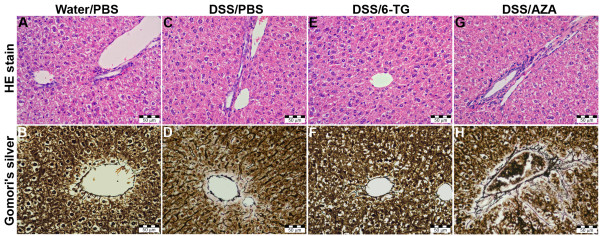
**Evaluation of liver toxicity by histology**. Normal structure is found in PBS-treated healthy (A and B), DSS/PBS-treated mice (C and D) and DSS/6-TG-treated (E and F), whereas a slight mononuclear infiltration and pericholangic sclerosis are seen in DSS/AZA-treated mice (G and H). (A, C, E and G: HE stain, B, D, F and H: Gomori's silver impregnation for reticulin).

The independent pathologists, unaware of prior treatment, observed evident pathological changes in most liver samples from the group of mice treated with 30 μg (6 out of 8 samples) and 60 μg (7 out of 8 samples) of AZA. All these samples showed pronounced fibrosis in the pericholangitic spaces and one sample displayed degenerative changes of hepatocytes (Figure 3).

Some discrete changes, such as slight fibrosis of pericholangitic space or sparse mononuclear cells in pericholangitic spaces were observed in mice treated with 10 μg (2 out of 8 samples), 20 μg (2 out of 8 samples) and 40 μg (1 out of 8 samples) of 6-TG and in one sample from the PBS treated mice. However, all these findings were considered within normal range. None of the evaluated samples showed signs of sinusoidal dilatation, veno-occlusive disease, NRH, steatosis, cirrhosis, necrosis of hepatocytes or cholestasis. The serum ALT activity was very low (ranging 20-30 U/l) in all groups (both treated and untreated), which indicate that no clinically significant hepatoxicity was induced in any group of mice (data not shown). The concentration of 6-TGN in red blood cells was below detection limit of 30 pmol/8*10^8 ^cells.

### Low-dose 6-TG is most effective in chronic colitis model

**Figure 4:**
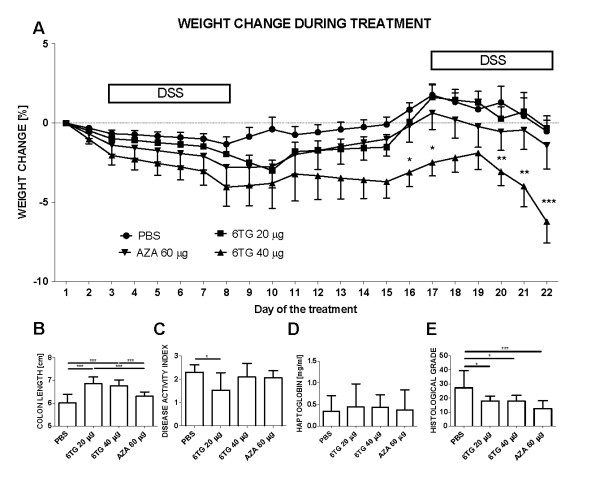
**Evaluation of treatment efficacy in chronic DSS-induced colitis**. Values are expressed as mean (dot or bar) ± SD (whisker). (A) Weight change during therapy. Body weight change was calculated for each mouse by dividing its body weight on the specified day by body weight at day 0 (body weight at the beginning of therapy) and expressed as percentage. (B) DAI (C) Colon Length (cm) (D) Serum haptoglobin concentration (mg/ml) and (E) Histological grade. The differences among experimental groups are analyzed with either two-way analysis of variance (ANOVA) with Bonferroni post-hoc test (Weight change; *P < 0.05; **P < 0.01; ***P < 0.001 vs. PBS-treated) or one-way ANOVA with Tukey's multiple comparison test (*P < 0.05; **P < 0.01; ***P < 0.001). Data are pooled from two independent experiments, each with 5 mice per group.

In the chronic colitis model, the therapeutic effect of 6-TG (both 20 and 40 μg/dose) expressed as prevention of colon shortening, was superior to either PBS (control) or AZA 60 (Figure 4B). However, due to the significant weight loss in mice with high-dose 6-TG administration, only the mice with low-dosed 6-TG administration shoved statistically significant improvement in disease activity index (Figure 4C). Interestingly, the weight loss in the 6-TG 40 treated group started to be statistically significant at the time of the 16^th ^dose of the 6-TG (Figure 4A). There were only minor signs (not significant) of colitis improvement in AZA 60 treated group and there were no statistically significant differences in serum haptoglobin concentration between the experimental groups (Figure 4D). Nevertheless, all thiopurine derivatives reduced mucosal damage (Figure 4E). Samples of liver after 22 thiopurine-doses were considered normal. None of the samples showed signs of sinusoidal dilatation, veno-occlusive disease, NRH, steatosis, cirrhosis, or necrosis of hepatocytes or cholestasis. Activity of ALT in serum was normal (between 20-30 U/l) and did not increase in any group throughout the whole treatment period, suggesting that there was no hepatotoxicity in any group (data not shown). The concentration of 6-TGN in red blood cells was below detection limit of 30 pmol/8*10^8 ^cells.

### 6-TG and AZA changes the cytokine production in the colon

**Figure 5:**
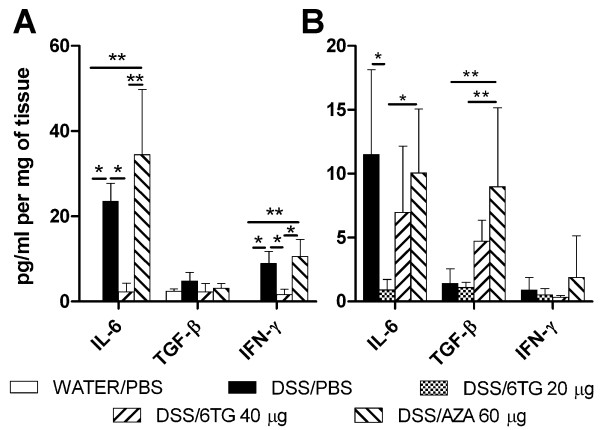
**Cytokine production in colon**. Cytokine production by colon tissue as analysed by tissue fragment culture and measured by ELISA in (A) Acute and (B) Chronic colitis model. Values are expressed as mean (bar) ± SD (whisker). The differences among experimental groups were analyzed with one-way analysis of variance (ANOVA) with Tukey's multiple comparison test (*P < 0.05; **P < 0.01). Data depict one out of two independent experiments, each with 5 mice per group.

There is a statistically significantly lower production of IL-6 and IFN-γ in DSS/6-TG 40 treated mice, as compared to DSS/PBS- or DSS/AZA 60 treated mice in the acute colitis model (Figure 5A). These changes in pro-inflammatory cytokines correlated with severity of colitis, which was much milder in DSS/6-TG treated animals. In the chronic colitis model, a decreased production of IL-6 in DSS/6-TG 20 treated mice as compared to either DSS/PBS- or DSS/AZA 60-treated mice was observed (Figure 5B). Production of IL-6 in DSS/6-TG 40 treated mice was intermediate between that of DSS/PBS- and DSS/6-TG 20 treated mice, the difference was not, however, significant. The production of TGF-β was higher in DSS/AZA treated mice as compared to either DSS/PBS- or DSS/6-TG 20 treated mice. Concentrations of IL-10 and TNF-α were low without any statistically significant changes. Neither 6-TG nor AZA changed cytokine production in non-colitic animals (data not shown).

### Apoptotic cells during thiopurine therapy

**Figure 6:**
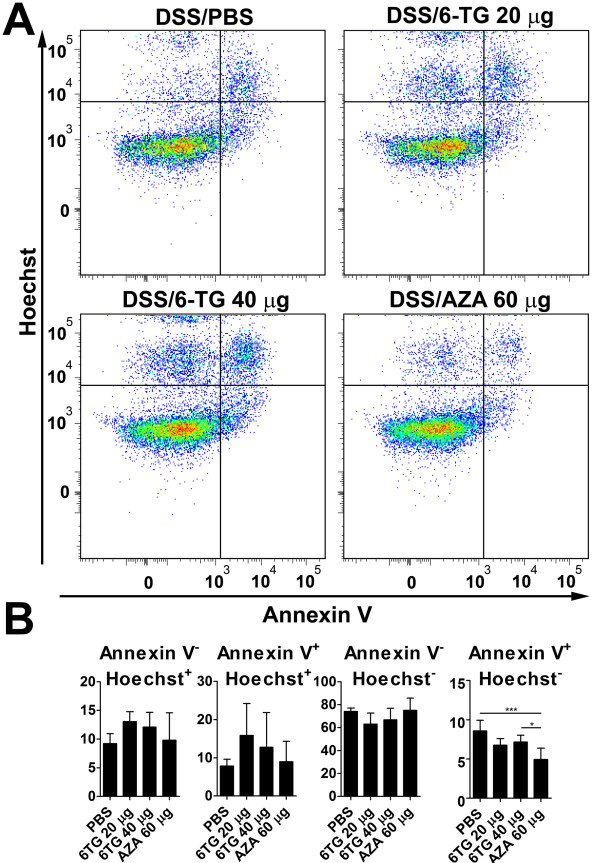
**The 6-TG increases the rate of apoptosis in T helper cells in model of chronic colitis**. (A) Typical plots depicting apoptosis in gated CD3^+^CD4^+^CD8^- ^T helper cells after the treatment of chronic colitis with PBS, 6-TG or AZA. (B) Graphs, mean (bar) ±SD (whisker), representing the relative proportions of CD3^+^CD4^+^CD8^- ^T helper cells in each quadrant. The differences among experimental groups were analyzed with one-way ANOVA with Tukey's multiple comparison test (*P < 0.05; ***P < 0.001).

There is no statistically significant effect on apoptosis *in vivo *during acute colitis. In chronic colitis, 6-TG induced apoptosis of Th (CD3^+^CD4^+^CD8^-^) cells while AZA did not. Early apoptotic Th (CD3^+^CD8^-^CD4^+^Annexin V^+^Hoechst^-^) cells were decreased in 6-TG-treated mice and even more in AZA-treated mice (Figure 6). A similar trend was seen in early apoptotic cytotoxic T (CD3^+^CD4^-^CD8^+^Annexin V^+^Hoechst^-^) cells (data not shown). AZA also decreased the proportion of early apoptotic cells (data not shown).

## Discussion

We have shown that 6-TG is effective in the treatment of colitis in mice, in a dose dependent manner, and that a dosage of 40 μg 6-TG per day (being 1.82 mg/kg) is superior to both AZA and placebo in acute experimental colitis therapy. Furthermore, in contrast to AZA, 6-TG therapy does not appear to induce histological liver damage. More precisely, slight portobiliary mononuclear infiltration with or without pericholangitic fibrosis (but always with well-defined interface) is an accidental finding in mice of the same breed, but damage to the adjacent reticulin was found only in mice that had AZA treatment. These results are in agreement with those of Petit et al., who showed that AZA and 6-MP decreases the viability of human hepatocytes even in clinically relevant concentrations when human treatment is considered, while 6-TG does not [24]. This difference in toxic effect among thiopurines could be explained by the production of toxic metabolites generated during the conversion of pro-drugs AZA and 6-MP to active metabolites 6-TGN, such as 6-methylmercaptopurines (6-MMP) which have been associated with liver test abnormalities [25-27]. In our experiments, AZA did not ameliorate acute colitis, as compared to 6-TG, which may be ascribed to differences in metabolization of thiopurine derivatives in mice as compared to humans. This may be also the reason behind our inability to detect 6-TGN in red blood cells of mice treated with thiopurine derivates.

Although all key enzymes are present in mice, it is difficult to compare mice pharmacology with that in human, which makes the comparison of dosages used in this experiment difficult compared to studies in humans. Nevertheless, the use of AZA in mice clearly resulted in histological liver abnormalities, while the use of 6-TG did not. Several studies about hepatological abnormalities induced by AZA or 6-MP have been recently published, which confirmed the results we observed in this mouse model [28-31].

The prevalence of NRH in the rest of the population without IBD is 2.6%, while it is 6% in the thiopurine-naïve IBD patients, 0-7% in IBD patients treated with low dose (approx. 20 mg/day) of 6-TG and in 18%-62% of patients with 40-80 mg/day of 6-TG [13,19,32,33]. These findings suggest that IBD itself and high dose of 6-TG, but not 6-TG in general, could be considered as a risk factor for NRH. The treatment duration should be also mentioned, because in human IBD, thiopurines are often administered for years, and although it was long enough to cause liver injury in AZA treated mice, nine days may not be a relevant time for induction of NRH.

Despite the fact that there were no signs of liver damage in chronic colitis, the mice with high dose of 6-TG had statistically significant decrease in body weight at the end of the experiment. This is in agreement with the fact that high doses of 6-TG can have general toxic effects, not related to the liver in these mice. We have not found any pathology in liver, lungs or bone marrow even with extreme dosage of 100 μg of 6-TG daily, however, we found hypocelularity in thymus and less activated follicles with wide mantle zones and arrest of haematopoiesis and deposition of hemosiderin in spleen (data not shown). These changes were mostly associated with high dose of 6-TG, and less in low dose of 6-TG and in AZA and could represent the consequence of the immunomodulatory properties of thiopurines.

We observed a decrease in the number of early apoptotic cells in the spleen of AZA-treated mice during the course of chronic colitis. This decrease was apparent in all thiopurine-treated geoups, when we gated to (CD3^+^CD4^+^CD8^-^) Th cells. Since the induction of apoptosis in activated Th cells is considered to be mechanism of thiopurine anti-inflammatory action *in vitro *[4,5], our findings suggest the existence of compensatory mechanisms *in vivo*.

Both 6-TG and AZA changed cytokine production in the colon of mice during inflammation. High levels of IL-6 and IFN-γ are produced in BALB/c mice during DSS-induced inflammation as we reported previously [22]. Since we observed a decrease in pro-inflammatory cytokines after 6-TG therapy in the acute and, to some extent, also in the chronic colitis model, this may corroborate the anti-inflammaory potential of thiopurine derivatives. Increase in TGF-β production in AZA-treated mice with chronic colitis could be caused by several mechanisms. TGF-β has many functions in gut mucosa homeostasis, it serves as growth factor for both epithelial and mesenchymal cells facilitating repair of mucosal injury and collagen deposition in IBD patients [34,35]. TGF-β has also important immunomodulatory functions, because it can dampen the inflammation by inducing Treg cells. In contrast to this, in a presence of high levels of IL-6, TGF-β might switch T cells to Th17 cells, thus promoting the protective immune response and inflammation [36,37].

Since the vast majority of AZA and 6-MP-intolerant IBD patients are able to tolerate maintenance treatment with 6-TG, the 6-TG was proposed as an alternative treatment of IBD in humans in case of intolerance or lack of therapeutic effect of AZA or 6-MP [10,14,18,38,39]. Our findings provide additional evidence that low dose 6-TG might still be considered as therapeutic option in those IBD patients who are intolerant or refractory to AZA or 6-MP.

## Conclusions

In conclusion, the use of 6-TG in the treatment of experimental colitis in mice appears superior to AZA administration and placebo. Beneficial effect of 6-TG treatment was associated with decrease in pro-inflammatory cytokines in colons of treated mice. Counterintuitively, the thiopurines decreased the number of early apoptotic T helper cells. Interestingly, in contrast to 6-TG, the use of AZA resulted in histological liver abnormalities.

## Abbreviations

IBD: inflammatory bowel disease; 6-TG: 6-tioguanine; AZA: azathioprine; DSS: dextran sulphate sodium; PBS: phosphate buffered saline; ALT: alanine aminotransferase; 6-MP: 6-mercaptopurine; 6-TGN: 6-thioguaninenucleotides; NRH: nodular regenerative hyperplasia; DAI: disease activity index; ANOVA: analysis of variance; 6-MMP: 6-methylmercaptopurines; HE: haematoxylin and eosin; ELISA: enzyme-linked immunoabsorbent assay

## Competing interests

The authors declare that they have no competing interests.

## Authors' contributions

MK, HTH, ML and CJM designed the study; MK, PR, KK and RMV performed experiments; MK, PR, BJ, NKB, AAB and RMV critically analyzed and interpreted data; MK, HTH, BJ and NKB wrote the manuscript and KK, AAB, ML and CJM revised it critically for important intellectual content. All authors read and approved the final manuscript.

## Pre-publication history

The pre-publication history for this paper can be accessed here:

http://www.biomedcentral.com/1471-230X/11/47/prepub
